# Traffic noise exposure impacts song production in wild male field crickets (*Gryllus bimaculatus*) under predator and intrasexual competition contexts

**DOI:** 10.1098/rsos.241346

**Published:** 2025-05-07

**Authors:** Mario Gallego-Abenza, David Wheatcroft

**Affiliations:** ^1^ Department of Zoology, Stockholm University, Stockholm, Sweden

**Keywords:** urbanization, anthropogenic noise, acoustic signals, intrasexual interactions, antipredator response, field crickets

## Abstract

Animals are known to adjust their acoustic signals in the presence of anthropogenic noise. These changes may affect fitness by altering susceptibility to predators or changing signal efficiency in intra- and intersexual interactions. Male field crickets, *Gryllus bimaculatus*, chirp to attract females, with chirp rate being an important success factor. Males reduce the chirp rate when exposed to predators or traffic noise and increase it in response to male competitors. However, the combined effects of these pressures on signalling are unknown. This study examined whether antipredator responses are influenced by male–male competition and variations in traffic noise exposure. We used substrate-borne vibrations to simulate a predator approaching and varied perceived male–male competition using playbacks to calling males. We found that responses to increased competition were affected by variation in traffic noise exposure, with males chirping faster as noise levels increased. Additionally, antipredator responses depended on an interaction between traffic noise and competition. Under high competition, males reduced the chirp rate as traffic noise increased. Our results demonstrate that adjustments in signal production in response to noise pollution may negatively impact communication in both antipredator and competitive contexts, indicating more pervasive effects of anthropogenic noise than previously recognized.

## 1. Introduction

Sexual signals produced to attract females can increase detection by predators [1,2]. When predation risk can be perceived by signallers, these may adjust their sexual signals to avoid detection [3–5]. For instance, under high predation risk, prey species tend to reduce the duration [6,7] or timing of courtship behaviours [8]. Additionally, the presence of competitors can lead to changes in signals [9,10]. At a finer scale, signals can be flexibly adjusted to the level of intrasexual competition, i.e. the number of rivals [11] or their perceived attractiveness [12]. In species where male–male competition involves physical encounters, increased male–male interactions can lead to a reduction in vigilance with a subsequent increase in predation risk [13,14]. As a response, competitors tend to engage in fewer aggressive interactions under experimentally high predation risk, as shown in Trinidadian guppy males [15]. Whereas long-term effects of predation pressure and competition shaping sexual signals are well understood [16,17], fewer studies have explored short-term plasticity of sexual signalling in response to these pressures acting in concert.

In the last decades, a novel, human-made stressor, anthropogenic noise, and its effects on animal acoustic communication have received increasing attention [18]. Anthropogenic noise has been reported to have negative effects in a broad variety of contexts. For instance, it can alter mating success [19,20], mate choice [21–23], predator–prey interactions [24,25], parent–offspring communication [26,27] and territorial display behaviour [28]. The signals of animals living in urbanized areas and experiencing chronic exposure to anthropogenic noise have been demonstrated to show a great variety of signal adjustments to improve transmission efficiency. Birds, for example, have been shown to alter the frequency, or pitch, of their vocalizations to avoid interference with anthropogenic noise [29,30] and to modify their singing activity patterns to avoid overlapping with noisy hours [31]. Although avian communication has received significant attention [32], adjustments to anthropogenic noise have also been described in other taxonomic groups, including mammals [33], fishes [34] and invertebrates [35,36]. More broadly, whether these adjustments in response to anthropogenic noise imply trade-offs in other contexts has received comparatively little attention [37]. For example, túngara frogs with urban-adapted acoustic signals attract more unintended parasites and predators if translocated to forest areas [38]. In this study, we examine: (i) the potential consequences of signal adjustment to anthropogenic noise on male–male competition; (ii) the interaction between predation risk and competition on acoustic signals in the presence of noise; and (iii) whether previous experience with anthropogenic noise may affect these relationships.

We chose wild field cricket males (*Gryllus bimaculatus*) to explore whether signal adjustments in response to traffic noise have maladaptive consequences on signalling behaviour in the presence of competitors and predators. *Gryllus bimaculatus* males can produce two distinct mating-related acoustic signals: (i) the calling song, a long-distance song elicited from natural shelters to attract phonotactic females [39]; and (ii) the courtship song, a short-distance signal given after females have approached the male [40,41]. Maintaining a fast calling song rate is essential to effectively attract a female [42]. However, long-distance calling songs may indirectly attract predators or parasitoids, as shown in other cricket species [2,43,44]. Field crickets have been shown to alter their calling song in response to traffic noise, predators, as well as the presence of competitors. For example, *G. bimaculatus* males reduce their chirp rate when exposed to passing cars. However, males located close to highways reduce their chirp rate less compared with individuals located further away from highways [45]. This reduced sensitivity to traffic noise may be owing to habituation to chronic exposure, consistent with the idea that reducing the chirping rate is maladaptive for sexual signalling because of the negative impacts on attracting a female. The perceived presence of predators is also known to reduce the calling song rate of crickets. For example, chirping in *Gryllus integer* is interrupted by an experimenter knocking on the lid of their cages, indicating their sensitivity to vibrational stimulations that could resemble an approaching predator [46]. Similarly, *G. bimaculatus* and *Gryllus campestris* males pause singing (up to 80 ms in *G. campestris*) when vibrationally disturbed [47]. The presence of competitors, by contrast, drives *G. bimaculatus* males to increase their chirp rate [48]. Thus, anthropogenic noise and predators have opposite impacts on male cricket calling song rate compared with competitors. Despite the fact that wild individuals living in urbanized environments are likely to experience some or all these factors at any given time, little is known about how *G. bimaculatus* males adjust their song in response to the interacting pressures of predation, intrasexual competition and traffic noise.

**Figure 1.**
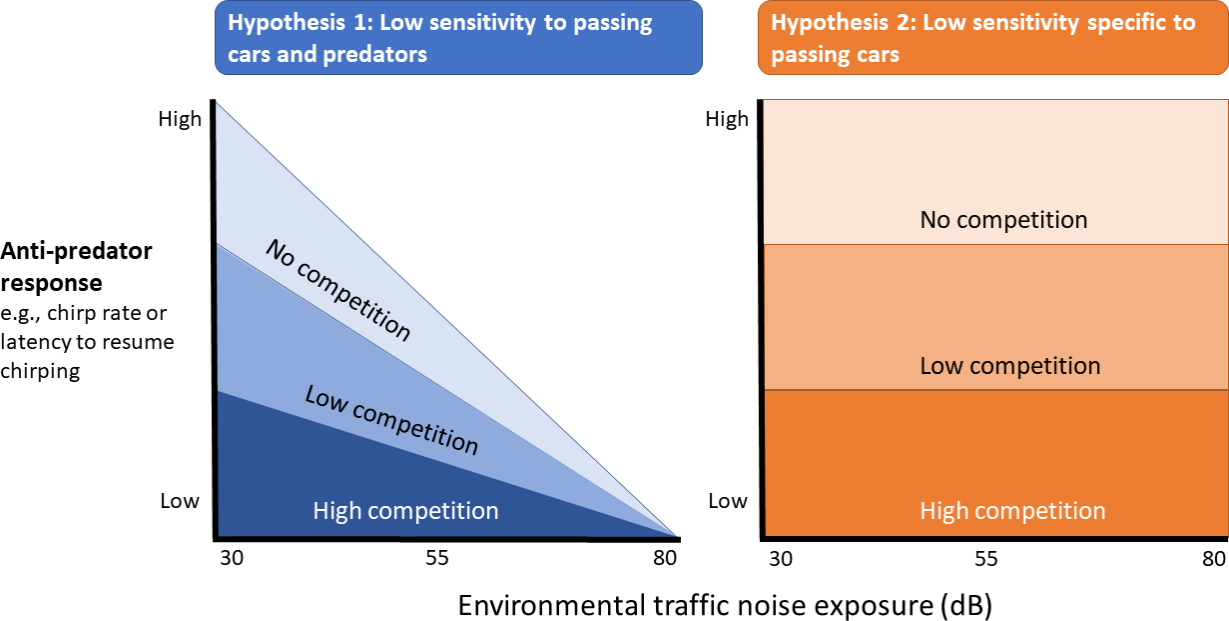
Predictions for anti-predator responses (measured as the alteration in calling song features) of field cricket males tested at different levels of both environmental traffic noise exposure and intrasexual competition. Whereas ‘low competition’ refers to the presence of one competitor, either a neighbour or the played-back male song, ‘high competition’ involves both competitors.

In this study, we test the hypothesis that natural variation in traffic noise exposure influences how wild field cricket *G. bimaculatus* males adjust their calling song in response to experimentally increasing perceived competition (playbacks of a calling male) and predation risk (vibrational disturbance). At baseline levels of predation risk (i.e. no vibrational disturbance), we predict all individuals to increase their chirp rate in response to increases in perceived competition, independently of the variation in traffic noise exposure. In addition, we test the hypothesis that typical antipredator responses of the species (e.g. reduction of chirp rate or increased latency to resume calling after disturbance) are impacted by the degree of competition. Based on the importance of producing an undisrupted chirp rate for attracting phonotactic females, we predicted that males being disturbed in the presence of competitors should return to baseline calling behaviour more quickly than males calling without competition. Finally, we explore how these factors act in concert by determining whether and how competition-dependent antipredator responses are influenced by an individual’s prior exposure to traffic noise. Prior exposure to traffic noise could influence responses to competitors and predators in a variety of ways. First, in addition to producing noise, passing cars generate substrate-borne vibrations that have been shown to hinder invertebrates that rely on such vibratory signals [49,50]. If crickets habituate to these vibrations, we predicted that males living near highways would have reduced antipredator responses, maintaining faster chirp rates after being threatened compared with males living in quiet environments. As a consequence, the effect of intrasexual competition on antipredator responses may be stronger in areas with low levels of traffic noise (see hypothesis 1 in figure 1). Alternatively, cricket males exposed to traffic noise might be able to discriminate between vibrations owing to passing cars and those owing to predators. In this case, we predict antipredator responses to be modulated by intrasexual competition, but not prior traffic noise exposure (see hypothesis 2 in figure 1).

## 2. Material and methods

### 2.1. Study site and species

This study took place in the Region of Murcia, a region in the southeast of Spain, where field crickets, *G. bimaculatus,* are commonly found in large meadows divided by highways. Field cricket males call from natural shelters to attract phonotactic females [39]. We tested 56 calling males located at different distances from three highways (RM-19 (37°51'37" N, 1°05'11" W), RM-1 (37°56'33" N, 0°57'15" W) and A−30 (37°52'55" N, 1°8'9" W)). Data collection occurred on nine nights with similar temperatures (range = 20–23°C) between 16 and 24 July 2021. Experiments were conducted between 22.30 and 03.15. Sampling of areas close to and far from highways, with decreasing and increasing traffic noise levels, respectively, was conducted at random times each night.

### 2.2. Experiment protocol

**Figure 2.**
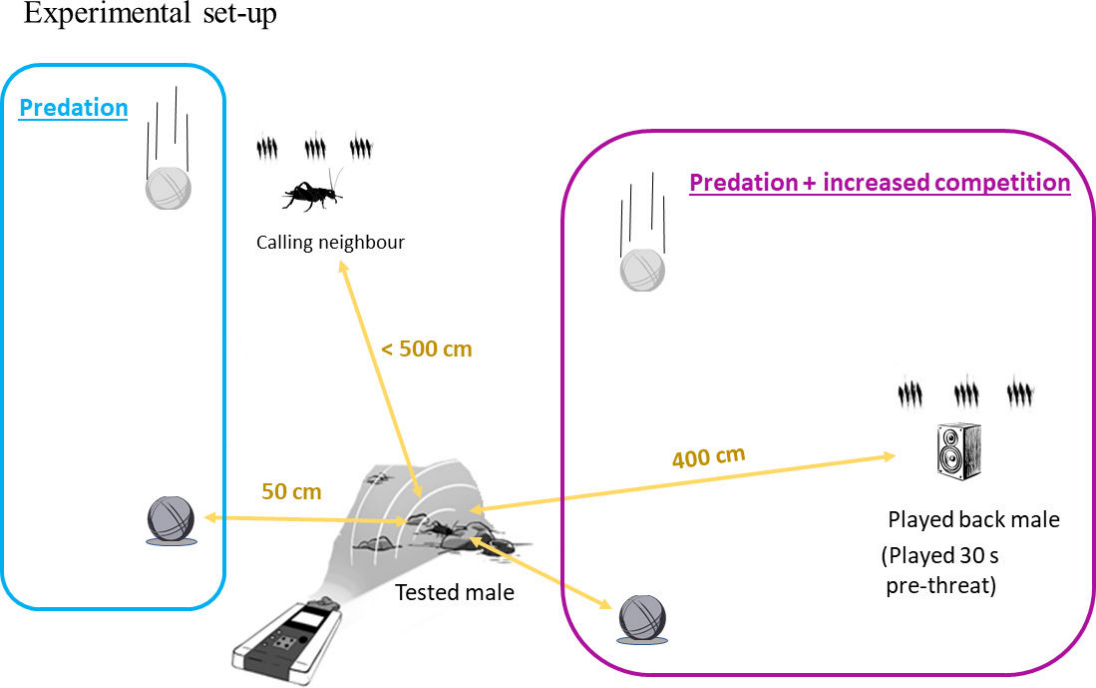
Schematic representation of the experimental set-up. The treatment refers to the two distinct scenarios in which tested males were threatened. In treatment predation, a dropping ball at a 50 cm distance from the tested male disrupts its calling behaviour. In the treatment predation and increased competition, the dropping ball is accompanied by a played-back calling song of a male, which starts 30 s before the ball is dropped. This allows us to take pre-threatening measurements while cricket males were exposed to a played-back male. The existence of a calling neighbour was reported when found within a 5 m radius distance from the tested male.

Each calling male was tested twice, experiencing thus two treatments: *predation*, where the presence of a predator was simulated, and *predation and increased competition*, where the presence of a predator was accompanied by a simulated competitor male within hearing distance (figure 2). The order of the treatments was randomized across tested males.

After a calling male was located on the ground by its calling song, the experimenter (M.G.-A.) approached within 1 m and remained motionless during the entire experiment. To simulate the approaching predator stimulus, we used two similar stainless metal balls (630 g) that were dropped independently (one for each treatment) on the ground from a height of 160 cm (figure 2). With the help of an L-shaped pole held in the air above the calling male, the experimenter estimated the distance of the two dropping points from the calling male, which was held consistent at 50 cm from the calling male. In the two treatments, dropping the ball occurred after males had resumed calling behaviour for at least 2 min of uninterrupted song (e.g. when the experimenter first approached or after the first treatment, following [45,51]). In the treatment *predation and increased competition*, we artificially increased intrasexual competition by broadcasting the chirping song of a conspecific male starting 30 s prior to the simulated predator. This allows us to directly measure the effect of intrasexual competition on singing behaviour. The loudspeaker (JBL Flip 4, frequency response = 70–20.000 Hz) was hung on a tripod at a height of 10 cm above the ground and pointed at the subject from a 4 m distance. The loudspeaker was placed when the experimenter first approached the subject. When a calling neighbour was heard, the loudspeaker pointed at the subject from a location opposite to the neighbour. The male song was played back using a digital music player (Musrun k188) at a standardized volume of 70 dBA, measured at 0.5 m of distance with a sound level meter (Radioshack, model 3300099, A-weighting, fast response). For playback recordings, we used three different males with little variation in chirp rate across them (range = 120–135 chirps min^−1^), which were recorded calling without nearby neighbours in the same area and from which we extracted a 1-min fragment, two and one individual were recorded in 2018 and 2021, respectively. Both tested and played-back males were recorded using a voice recorder (Olympus DM670, sampling rate = 44.1 Hz, 16-bit, WAV format) placed on the ground at approximately 50 cm distance (figure 2). In the case of tested individuals, recordings began right after placing the recorder on the ground and included both pre- and post-threat songs. Once the experiment concluded, we collected the global positioning system (GPS) coordinates of the male’s location using the app UTM Geo Map, installed on a smartphone Xiaomi Redmi 9. We noted whether there was a calling neighbour male within a 5 m radius using a mechanical tape measure. The 5 m threshold is supported by research studies on wild field crickets, indicating the lack of response to competitors calling from a larger distance [39,52,53]. Finding a chirping neighbour within such distance constituted the binomial variable, hereafter called *neighbour presence* in our statistical analyses. Out of 56 tested males, 22 had a nearby calling neighbour during testing. Thus, whereas individuals calling in the absence of a neighbour shifted from no intrasexual competition to low (one competitor; played-back male), a subset of field cricket males experienced a transition from low (one competitor; neighbour) to high competition (two competitors; neighbour and played-back male).

### 2.3. Acoustic analyses

First, we calculated the distance to the highway for each tested male cricket based on his GPS coordinates using a built-in tool in Google Earth software. We transformed distances to the highway asphalt (range = 3–814, average = 189.23 m) into an individual value of *environmental traffic noise exposure* (dBA) (range = 38.8–74.3, average = 48.2 dBA) based on a gradient of traffic noise attenuation previously established in the same area by us. In that study, we created a gradient by reporting the average of the peaks of noise (dBA) of three passing cars measured at different distances to the highway, from 3 to 225 m. We fitted an exponential decay curve to the noise–distance gradient. Using the coefficients from this model, we used an individual’s distance to the highway to estimate their environmental traffic noise exposure [45]. We used Raven Pro 1.5 [54] to visualize and analyse the acoustic features of focal males calling songs on the spectrogram (fast Fourier transform size = 512, window function = Hann, frequency resolution of 124 Hz and temporal resolution of 11.6 ms). First, we measured the latency (in seconds) it took for males to resume calling (i.e. the first elicited chirp) after being threatened by dropping balls. Likewise, we measured the silence gap between successive chirps to assess variation inter-chirp interval (ICI, as a measurement of chirp rate). Specifically, we measured the last 10 ICIs before the calling song was interrupted (pre-threat ICI, hereafter named *baseline ICI*) and the 10 first ICIs after resuming calling (post-threat ICI). In total, we took 40 measurements per individual (10 of baseline and 10 of response for each of the treatments: *predation* and *predation & increased competition*). We reported the number of pulses per chirp of each measurement, allowing us to model both *baseline pulses per chirp* and *delta pulses per chirp*. Analysing baseline measurements themselves allowed us to investigate whether *environmental traffic noise exposure* and/or *neighbour presence* influence how crickets respond to increased intrasexual competition via playback of another male’s song (i.e. the baseline measurements from the *predation and increased competition* treatment). Dropping balls occurred in silence gaps between passing cars. We felt justified in taking 10 measurements before and after each treatment owing to our interest in recording the immediate response to the treatments and to minimize the potential confounds of real cars passing by during the experiments.

### 2.4. Statistical analyses

We used R software (v. R. 4.2) [55] to perform our statistical analyses. We first assessed the effectiveness of the experimental vibrational disturbance on chirp rate interruption. To do this, we ran a *t*‐test comparing the average of the 10 *baseline ICI* from the treatment *predation* (no played-back male involved) with the *latency to resume calling* after threatening. To conduct further statistical analysis on *latency to resume calling* (seconds), we noticed that it violated parametric assumption and we transformed it into milliseconds by multiplying it by 1000. This resulted in a discrete variable response following a negative binomial distribution (estimated using the function ‘descdist’ in the package ‘fitdistrplus’ v. 1.1.8 [56]). We used 10 ICIs before each predator simulation (pre-threat songs, i.e. *baseline ICI*) to model the responses of crickets to increased competition in a non-predator context. To assess variation in chirp rate after predator simulation, we calculated 10 *delta ICI* measurements by subtracting the average of treatment-specific *baseline ICI* to the 10 measurements of the response (post-threat) period. Because the resulting *delta ICI* did not follow a normal distribution, we transformed it into a discrete variable by multiplying by 10 000 and adding 4197 to only contain positive values. *Delta ICI* was determined to follow a negative binomial distribution using the function ‘descdist’. We thus evaluated three separate generalized linear mixed models (GLMMs) explaining variation in *latency to resume calling*, *baseline ICI* and *delta ICI*, using the function ‘glmmTMB’ within the package ‘glmmTMB’ v. 1.1.4 [57]. For each model, explanatory variables were *treatment order* and a three-way interaction term consisting of the z-transformed *environmental traffic noise exposure (dB*), *treatment* (*predation* and *predation and increased competition*) and *neighbour presence* (*yes* or *no*). This three-way interaction term allowed us to assess whether different levels of intrasexual competition (one competitor, either a neighbour or played-back male or both of them) had an effect on the calling behaviour of cricket males experimentally threatened at different traffic noise exposure. To estimate the significance of differences between trends resulting from the three-way interaction term, we conducted *post hoc* analyses using the function ‘emtrends’ within the package ‘emmeans’ v. 1.8.1.1 [58]. While these pairwise comparisons are reported in main text tables, full model results can be found in the electronic supplementary material. *Played-back male ID* and *tested male* nested within *highway ID* were included in all models as random factors. We validated the inclusion of the significant three-way interaction term through the likelihood ratio test, function ‘lrtest’ within the package ‘lmtest’ v. 0.9.40 [59] with their respective models containing the three two-way interaction terms, a single two-way interaction term, non-interacting terms and null models. A similar modelling approach was used to explain the variation in both *baseline pulses per chirp* and *delta pulses per chirp* (Poisson distribution) and its results are placed in the electronic supplementary material owing to the lower observed effect of the predictors of interest.

## 3. Results

### 3.1. As traffic noise increases, cricket males chirp at faster rates when experiencing increased intrasexual competition

#### 3.1.1. Baseline inter-chirp interval

**Figure 3.**
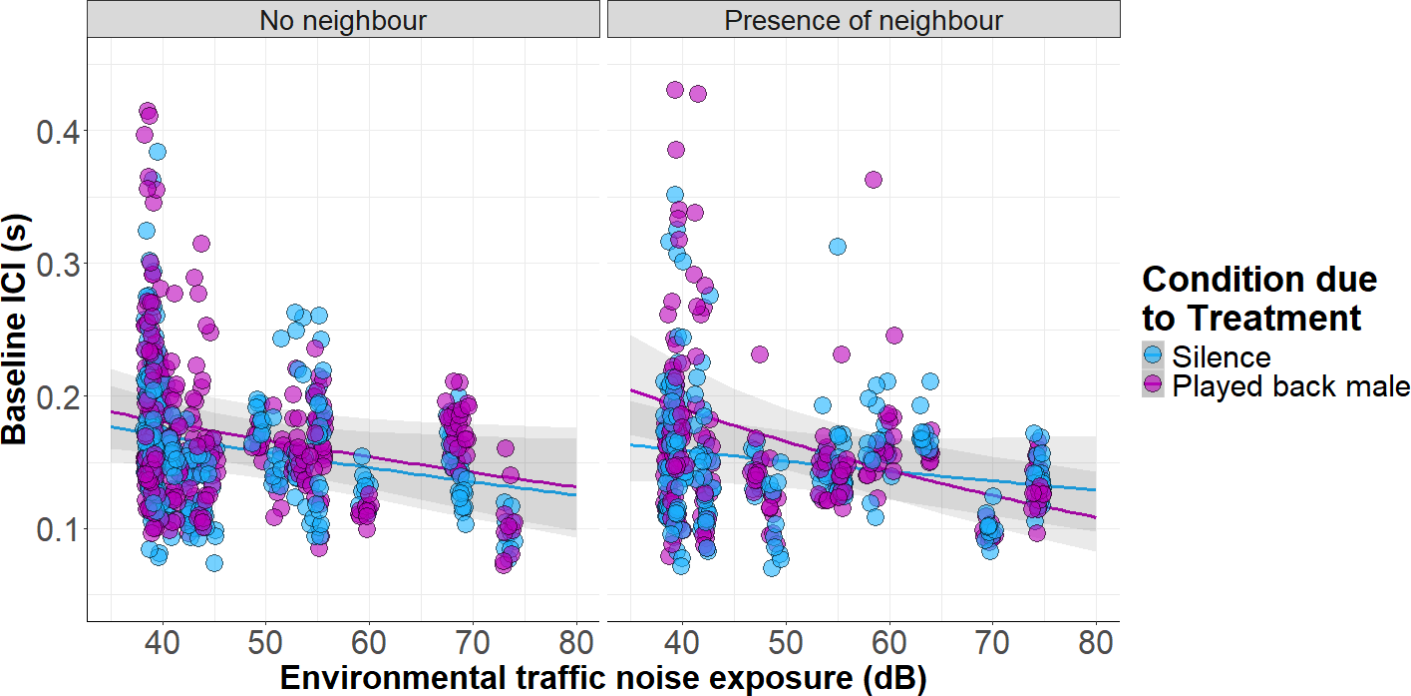
Baseline ICI measurements of cricket males found calling at different levels of environmental traffic noise with and without a neighbour within audible distance and experimentally exposed to increased intrasexual competition. Predicted data were extracted from the model containing the three-way interaction term with the function ‘ggpredict’, R package ‘ggeffects’ v. 1.1.4 [60]. Raw points are width-jittered for better visualization and eight points above 0.5 are not displayed, five of them belong to the ‘presence of neighbour’ panel.

Overall, baseline measurements (prior to threatening) revealed that cricket males living close to highways chirp with lower ICI, i.e. faster chirp rate (see table 1a and figure 3). Interestingly, this negative correlation is more pronounced at the highest level of intrasexual competition (model containing the three-way interaction between *environmental traffic noise exposure (dB*), *treatment* and *neighbour presence* compared with a model containing the three terms not interacting: likelihood-ratio test, $X_{4}^{2}$ = 17.165, *p*-value = 0.0018). The various model comparisons are: model comparison with a model containing the two-way interaction term ‘*environmental traffic noise exposure (dB) × treatment*’: likelihood-ratio test, $X_{3}^{2}$ = 10.128, *p*-value = 0.0175; model comparison with a model containing the two-way interaction term ‘*environmental traffic noise exposure (dB) × neighbour presence*’: likelihood-ratio test, $X_{3}^{2}$ = 17.049, *p*-value = 0.0007; and model comparison with a model containing the two-way interaction term ‘*treatment × neighbour presence*’: likelihood-ratio test, $X_{3}^{2}$ = 16.181, *p*-value = 0.001. *Post hoc* analyses revealed that as traffic noise increases, crickets showed a significant reduction of ICI (chirped faster) when transiting from low to high competition (*p*-value = 0.0001, table 1c). By contrast, we found no effect of the playback on the responses of males without neighbours (*p*-value = 0.8801, see table 1c).

**Table 1.** Post hoc test of the three-way interaction term: environmental traffic noise exposure × treatment (silence, played-back male) × neighbour presence (yes/no) using the ‘emtrends’ function. (The results show (a) how traffic noise exposure modulates baseline ICI in each of the four different combinations, (b) a pairwise comparison of these trends testing for the presence of a neighbour in each of the treatments, and (c) a pairwise comparison to test whether trends differ between treatments across the two scenarios: neighbour (no) and neighbour (yes). Bold *p*-values indicate a significance of <0.05.)

response variable: baseline ICI (inter-chirp interval)					
fixed variable: environmental traffic noise exposure (dB)					
a)	estimate ± s.e.	d.f.	lower CI	upper CI	*P-*value
*contrast*: silence					
neighbour presence (no)	−0.0076 ± 0.0039	1107	−0.0153	7.35 × 10^-5^	0.052
neighbour presence (yes)	−0.0052 ± 0.004	1107	−0.0131	2.75 × 10^-3^	0.199
*contrast*: played back male					
neighbour presence (no)	−0.0079 ± 0.0039	1107	−0.0156	−2.55 × 10^-4^	**0.043**
neighbour presence (yes)	−0.0141 ± 0.004	1107	−0.0220	−6.17 × 10^-3^	**0.0005**

b) pairwise comparison	estimate ± s.e.	d.f.	*t*	*P*-value	
*contrast*: silence					
neighbour presence (no – yes)	−0.0024 ± 0.0056	1107	−0.434	0.6643	
*contrast*: played back male					
neighbour presence (no – yes)	0.0061 ± 0.0056	1107	1.094	0.2743	

c) pairwise comparison	estimate ± s.e.	d.f.	*t*	*P-*value	
*contrast*: neighbour presence *no*					
silence – played back male	0.0003 ± 0.002	1107	0.151	0.8801	
*contrast*: neighbour presence *yes*					
silence – played back male	0.0089 ± 0.0022	1107	4.02	**0.0001**	

### 3.2. Calling song is interrupted by simulated approaching predators

#### 3.2.1. Latency to resume chirping

The baseline ICI of males chirping in the absence of a played-back male was 0.159 s on average (s.e.m. = ± 0.0048, *n* = 56). When exposed to a simulated predator, males on average cease calling after 14.811 s (s.e.m. = ± 3.357, *n* = 56), showing a significant reduction in chirping rate (paired *t*‐test: *t*_55_ = −4.36, *p-*value ≤ 0.0001). Thus, dropping balls at 50 cm from the subjects created enough vibrational disturbance to instigate an antipredator response. Neither traffic noise exposure nor the degree of competition affected the latency to resume calling after being threatened (electronic supplementary material, table S4). However, cricket males resumed calling more quickly when threatened in the second trial, suggesting habituation to the simulated predator (estimate = −0.329, s.e. = 0.162, Z = −2.03, *p* = 0.042; see the electronic supplementary material, table S4).

### 3.3. Significant reduction of chirp rate when threatened under high compared with low intrasexual competition observed in noisy areas

#### 3.3.1. Delta inter-chirp interval

**Figure 4.**
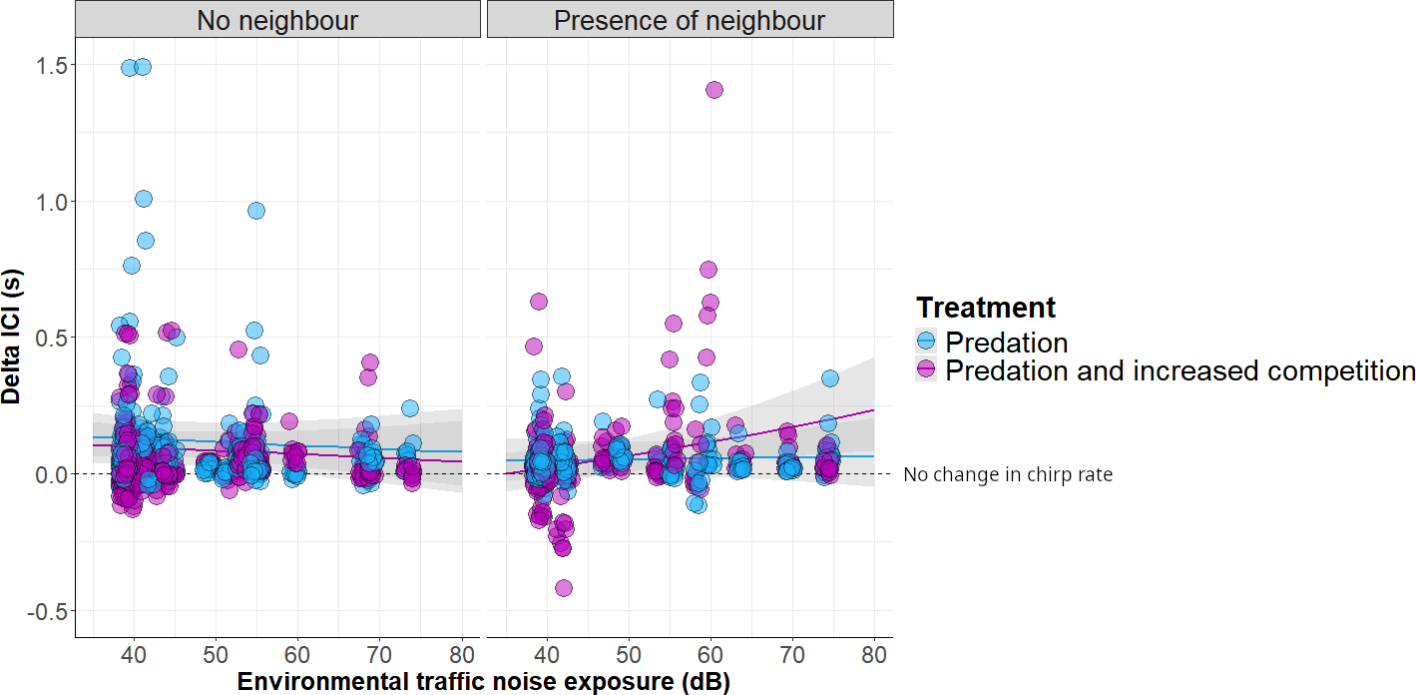
Delta ICI of experimentally threatened calling males found at different levels of environmental traffic noise without (left) and with a neighbour (right) and experimentally exposed to increased intrasexual competition (treatment: predation and increased competition). Predicted data were extracted with the function ‘ggpredict’ from the model containing the three-way interaction. Raw data are width-jittered for better visualization and 14 points above 1.5 s are not displayed. Nine belong to the ‘no neighbour’ panel (five and four from predation and predation and increased competition, respectively). The five non-displayed points from the presence of the neighbour panel belong to the treatment *predation and increased competition*. Positive delta ICI values above the dashed line indicate a reduction in chirp rate.

Overall, cricket males reduced their chirp rate after being threatened (*delta ICI*: mean = 0.0998, range = −0.4197to 9.3136 s). However, this response was influenced by an individual’s exposure to traffic noise as well as by the level of intrasexual competition. Model comparisons support the inclusion of the significant three-way interaction term comprising *environmental traffic noise exposure, treatment and neighbour presence* over the model containing the non-interacting terms: likelihood ratio test, $X_{4}^{2}$ = 16.47, *p*-value = 0.0024. The various model comparisons are: model comparison with a model containing the two-way interaction term ‘*environmental traffic noise exposure (dB) × treatment*’: likelihood-ratio test, $X_{3}^{2}$ = 11.123, *p*-value = 0.0111; model comparison with a model containing the two-way interaction term ‘*environmental traffic noise exposure (dB) × neighbour presence*’: likelihood-ratio test, $X_{3}^{2}$ = 14.157, *p*-value = 0.0027; model comparison with a model containing the two-way interaction term ‘*treatment × neighbour presence*’: likelihood-ratio test, $X_{3}^{2}$ = 12.467, *p*-value = 0.0059. The presence of a natural competitor (with neighbour presence) resulted in a significant effect of the played-back male song on *delta ICI*: as traffic noise increases, the resumed chirp rate was significantly slower compared with when resuming chirping without an additional competitor (see table 2c; figure 4).

**Table 2.** Post hoc test of the three-way interaction term: environmental traffic noise exposure × treatment (predation/predation and increased competition) × neighbour presence (yes/ no) using the ‘emtrends’ function. (The results show (a) how traffic noise exposure modulates delta ICI in the four scenarios of intrasexual competition determined by having or not having a neighbour and the treatment, (b) a pairwise comparison of these trends testing for the presence of a neighbour in each of the treatments, and (c) a pairwise comparison to test whether trends differ between treatments across the two scenarios: neighbour (no) and neighbour (yes). Bold *p*-values indicate a significance of <0.05.)

response variable: delta ICI (inter-chirp interval)					
fixed variable: environmental traffic noise exposure (dB)					
a)	estimate ± s.e.	d.f.	lower CI	upper CI	*P-*value
*contrast*: predation					
neighbour presence (no)	−0.0024 ± 0.0037	1107	−0.0099	0.0049	0.5181
neighbour presence (yes)	0.0007 ± 0.0037	1107	−0.0066	0.0081	0.8476
*contrast*: predation & increased competition					
neighbour presence (no)	−0.0027 ± 0.0038	1107	−0.0102	0.0046	0.4638
neighbour presence (yes)	0.0098 ± 0.0038	1107	0.0023	0.0174	**0.0105**

b) pairwise comparison	estimate ± s.e.	d.f.	*t*	*P*-value	
*contrast*: Predation					
neighbour presence (no - yes)	−0.00318 ± 0.00534	1107	−0.596	0.5515	
*contrast*: predation & increased competition					
neighbour presence (no - yes)	−0.01265 ± 0.00539	1107	−2.344	**0.0192**	

c) pairwise comparison	estimate ± s.e.	d.f.	*t*	*P*-value	
*contrast*: neighbour presence *no*					
predation - predation & increased competition	0.00033 ± 0.00264	1107	0.125	0.9006	
*contrast*: neighbour presence *yes*					
predation - predation & increased competition	−0.00914 ± 0.00287	1107	−3.186	**0.0015**	

### 3.4. Baseline pulses per chirp and delta pulses per chirp

*Baseline pulses per chirp* were influenced neither by traffic noise nor intrasexual competition. In the predator context, *delta pulses per chirp*, our results suggested a tendency for the threatened highway-dwelling individuals to maintain a similar number of pulses compared to baseline at the highest level of intrasexual competition. Detailed information regarding how the number of pulses per chirp changes prior to threatening (*baseline pulses per chirp*) and post-threatening (*delta pulses per chirp*), can be found in the electronic supplementary material, table S3 and tables S5 and S6 respectively.

## 4. Discussion

In this study, we first demonstrated that environmental traffic noise affects field cricket songs under different levels of intrasexual competition. In a non-predator context, as the environmental traffic noise increases (i.e. closer to the highway), cricket males increase their chirp rate when experiencing the highest level of intrasexual competition (simulated intruder and neighbour, hence two competitors) compared with lesser competition (neighbour). Previous studies demonstrated that laboratory-reared *G. bimaculatus* males increase their chirp rate in the presence of a competitor [48]. Our results in the non-predator context indicate that such competition-dependent increases in chirp rate were more pronounced in wild males that already had a nearby calling competitor and were living near roads. When these males were experimentally exposed to a simulated predator, we found that the features of their resumed song depended on an interaction between environmental traffic noise exposure and intrasexual competition. In particular, highway-dwelling males experiencing the highest level of competition reduced their chirp rates after being exposed to vibrations simulating a predator. Thus, the significant increase in the chirp rate observed in these males when exposed to a second competitor was no longer maintained in their song after being threatened.

We here outline three alternative explanations for our shown results in both non-predator and predator contexts. First, one would hypothesize that wild populations are structured according to the gradient of traffic noise and age. *Gryllus bimaculatus* males gradually reduce their chirp rate as they age, mainly owing to the degradation of stridulation muscles [42,61]. Although we did not estimate the age of the tested males, the observed tendency of males living closer to roads to chirp faster during baseline (figure 3) would be consistent with the idea that younger individuals settled to signal closer to the roads. If this is the case, the increase in the chirp rate when hearing a second competitor could be explained by a higher muscular strength and, therefore, is a physiological capability only shown in young individuals. However, going against this idea, *G. bimaculatus* males tend to be sedentary, with short movements over the range of a few metres where they can dispute for occupied burrows with other males [62,63]. In our study, the distances to the asphalt at which tested males were found ranged from 3 to 814 m, making the relationship between age and road distance unlikely. A second explanation for why highway-dwelling crickets respond stronger (i.e. increasing chirp rate) to a second competitor is that they must effectively exploit silence gaps between passing cars, and, thus, are particularly sensitive to competitors during any period of silence. Such exploitation of silence windows may be supported by the findings on females’ phonotactic behaviour failing to locate calling males under traffic noise [23]. Mate choice based on courtship song was similarly affected by both traffic and white noise conditions [21]. These findings underlie the low efficacy of our study species’ signals under controlled conditions. However, little is known about how the fluctuant character of traffic noise, defined by peaks of amplitude, modulates females’ mate searching behaviour in the wild. Females’ phonotactic behaviour may occur intermittently, with micro-pauses during high peaks of noise. These factors reinforce the idea of traffic noise heightening the effect of new arriving competitors and subsequent song adjustments in limited time windows in which signalling is effective. A third possibility is that males from quiet areas adopted an alternative mating strategy, e.g. reduced signalling effort in the presence of competitors, as observed in species where males form leks. For instance, in desert grasshoppers, *Ligurotettix coquilletti*, acoustically inactive males are more likely to be found at higher population densities [64]. In other species of field crickets, non-singing males can be found allocated near callers to intercept their search for actively calling mates [65]. *Gryllus bimaculatus* males can form calling aggregations in the wild, which are thought to increase female attraction [39]. Under controlled conditions, males of lower competitive abilities tend to reduce their calling effort, measured as calling bouts, in higher densities [62]. Whereas we showed that the likelihood of having a neighbour was not explained by the environmental traffic noise gradient (see the electronic supplementary material, table S7), our results indicate that only those individuals far from the road would seemingly benefit from the artificially formed lek. We then suggest that highway-dwelling males have heightened sensitivity to competitors in silence windows near the roads as an adaptation to cope with competitors under traffic noise. Laboratory experiments in which both intrasexual competition and noise exposure are controlled could help to understand the adaptative use of silence windows in male–male signalling competition.

Interestingly, the exposure to a simulated predator alters the effect that traffic noise and competition have on the chirp rate. Highway-dwelling males experiencing high levels of competition resume calling after being threatened with a relatively lower chirp rate compared with their baseline than males living far from the highways. These results on chirp rate go counter to our two hypotheses. The unexpected reduction of chirp rate in highway-dwelling crickets does not align with hypothesis 1, where habituation to passing cars would have implied habituation to other vibratory disturbances. Neither do these results follow hypothesis 2, where the extent to which song adjustments after disturbance would have been driven specifically by the level of intrasexual competition regardless of traffic noise exposure (see figure 1). Based on the observed trends, hypothesis 2 would thus explain responses in areas with low levels of environmental traffic noise, where the presence of a second competitor may lead to a similar chirp rate after being threatened (see figure 4). The reduced chirp rate shown by highway-dwelling males when hearing two competitors leads us to propose a shift in mating strategy depending on both predation risk and intrasexual competition. Thus, in noisy areas, crickets under predation risk may rely on others for female attraction and, thereby, reduce signalling effort. From the receivers’ perspective, little is known about the negative effect of vibrational disturbances on females’ phonotactic behaviour. Assuming predator-driven pauses made by nearby phonotactic females, a post-threat reduced chirp rate would ultimately imply reduced costs for threatened males located near the roads. Interestingly, the tendency of males located in noisy areas to resume calling with lesser reduced chirps in pulses relative to males far from highways seems to follow hypothesis 1 (see the electronic supplementary material, table S6). However, the number of pulses per chirp has been shown to be less relevant for females than the chirp rate, where females still respond to songs that contain a moderate ratio of *odd* chirps within a calling song [66].

This study shows the importance of investigating the multi-context efficiency of acoustic signals that are adjusted in the presence of noise. We demonstrate that evaluating competitors in the presence of a predator depends on prior traffic noise exposure and might have consequences on individual fitness by affecting female choice. Further studies, including (i) body size, as a proxy for male–male competitive ability [62], (ii) age determination, to investigate a potential dispersion of younger males towards noisy areas, and (iii) boldness tests of wild-caught individuals, to examine a potential behavioural syndrome linking boldness responses to both competitors and predators, could help us to understand the observed distinct ways in which crickets value competitors along the natural variation of traffic noise exposure. Additionally, monitoring the daily patterns of overall calling effort related to experienced traffic noise might shed light on how crickets adapt their song as antipredator responses. Crickets from noisy areas may invest more calling effort in male–male interaction after rush hours when they are more likely to be assessed and located by females. Although this study took place in homogeneous areas (i.e. flat agriculture fields lacking any vegetation when experiments were conducted), we cannot rule out the fact that other factors apart from traffic noise (e.g. lights of passing cars or human activity) may influence the responses observed here in cricket males. Additional studies involving the translocation of individuals in the field or isolating the effect of noise under laboratory conditions would help to identify the noise-specific effects on insect behaviour. This study shows that neither the complexity of urbanization and its multi-modal effects on biodiversity nor the variety of fine-scale behavioural adaptations that enable species persistence in a changing world are well understood.

## Data Availability

Dataset and R code are available in the following link [67]. Supplementary material is available online [68].
